# Aberrant GABA_A_ Receptor-Mediated Inhibition in Cortico-Thalamic Networks of Succinic Semialdehyde Dehydrogenase Deficient Mice

**DOI:** 10.1371/journal.pone.0019021

**Published:** 2011-04-19

**Authors:** Adam C. Errington, K. Michael Gibson, Vincenzo Crunelli, David W. Cope

**Affiliations:** 1 School of Biosciences, Cardiff University, Cardiff, United Kingdom; 2 Department of Biological Sciences, Michigan Technological University, Houghton, Michigan, United States of America; National Institute on Aging Intramural Research Program, United States of America

## Abstract

Aberrant γ-aminobutyric acid type A (GABA_A_) receptor-mediated inhibition in cortico-thalamic networks remains an attractive mechanism for typical absence seizure genesis. Using the whole-cell patch clamp technique we examined ‘phasic’ and ‘tonic’ GABA_A_ inhibition in thalamocortical neurons of somatosensory (ventrobasal, VB) thalamus, nucleus reticularis thalami (NRT) neurons, and layer 5/6 pyramidal neurons of the somatosensory (barrel) cortex of succinic semialdehyde dehydrogenase (SSADH) knock-out (SSADH^−/−^) mice that replicate human SSADH deficiency and exhibit typical absence seizures. We found increased sIPSC frequency in both VB and NRT neurons and larger sIPSC amplitude in VB neurons of SSADH^−/−^ mice compared to wild-type animals, demonstrating an increase in total phasic inhibition in thalamus of SSADH^−/−^ mice. mIPSCs in both VB and NRT neurons were no different between genotypes, although there remained a trend toward more events in SSADH^−/−^ mice. In cortical layer 5/6 pyramidal neurons, sIPSCs were fewer but larger in SSADH^−/−^ mice, a feature retained by mIPSCs. Tonic currents were larger in both thalamocortical neurons and layer 5/6 pyramidal neurons from SSADH^−/−^ mice compared to WTs. These data show that enhanced, rather than compromised, GABA_A_ receptor-mediated inhibition occurs in cortico-thalamic networks of SSADH^−/−^ mice. In agreement with previous studies, GABA_A_ receptor-mediated inhibitory gain-of-function may be a common feature in models of typical absence seizures, and could be of pathological importance in patients with SSADH deficiency.

## Introduction

Succinic semialdehyde dehydrogenase (SSADH) deficiency is an autosomal recessively inherited disorder which, when compared with other neurometabolic disorders, is relatively common with approximately 400 identified cases worldwide [1]. Loss of SSADH activity compromises GABA degradation, leading to the accumulation not only of GABA but also of γ-hydroxybutyric acid (GHB) in the cerebrospinal fluid [1], [2]. Clinical symptoms of SSADH deficiency are variable, but typically include delayed intellectual, speech and language development, hypotonia, ataxia, sleep disturbances and an array of epilepsies, including generalized tonic-clonic, absence and myoclonic seizures [3]–[5]. Recently, SSADH deficient mice were developed that replicate the GHB and GABA accumulation exhibited in humans, and have a strikingly similar epileptic phenotype [1], [6], [7]. In particular, homozygous SSADH knock-out (SSADH^−/−^) mice display typical absence seizures that appear at the beginning of the third postnatal week, evolve into myoclonic and generalized convulsive seizures, and finally progress to lethal status epilepticus [6], [8], [9]. These mice are therefore a valuable tool in examining the pathological cellular mechanisms underlying seizure genesis in SSADH deficiency.

Typical absence seizures characterize many idiopathic generalized epilepsies and are generated in cortico-thalamic networks [10], [11]. In rodent models, spike-and-wave discharges, the EEG hallmark of absence seizures, arise in layer 5/6 neurons of the somatosensory cortex and propagate to the underlying thalamus [12]–[14]. However, recruitment of thalamocortical neurons of the somatosensory ventrobasal (VB) nucleus and neurons of the nucleus reticularis thalami (NRT) is required for the full electrographic and behavioural expression of seizures [13]. Whilst compromised GABAergic inhibition in cortico-thalamic networks is an attractive pathological mechanism for seizure genesis [15], [16], we recently demonstrated that extrasynaptic GABA_A_ receptor-mediated inhibition is increased in thalamocortical neurons from multiple and diverse models of absence seizures, and that extrasynaptic GABA_A_ receptor hyperfunction in the thalamus is critical for seizure genesis [17]. Extrasynaptic GABA_A_ receptors generate a distinct type of inhibition from their synaptic counterparts. Synaptic GABA_A_ receptors are activated by vesicular GABA release from GABAergic terminals and generate classical ‘phasic’ inhibitory post-synaptic currents (IPSCs). By comparison, extrasynaptic GABA_A_ receptors are activated by spillover of GABA from the synaptic cleft and generate a persistent or ‘tonic’ GABA_A_ current [18], [19]. Previous studies in SSADH^−/−^ mice have observed altered phasic and tonic GABA_A_ inhibition in hippocampal CA1 pyramidal and cortical layer 2/3 pyramidal neurons [20]–[22], but whilst these findings may have relevance for the development of myoclonic and generalized convulsive seizures, GABA_A_ receptor-mediated inhibition has not been examined in neurons that actively participate in the generation of typical absence seizures, i.e. thalamocortical neurons, NRT neurons and layer 5/6 pyramidal neurons. We have therefore examined phasic and tonic GABA_A_ currents in thalamic and cortical neurons from SSADH^−/−^ mice and compared them to wild-type (WT) littermates. Our data demonstrate altered GABA_A_ receptor-mediated inhibition in all three neuron types that may underlie the appearance of absence seizures in SSADH^−/−^ mice and be of pathological importance in human SSADH deficiency.

## Methods

All animal procedures were carried out in accordance with local ethical committee guidelines (Cardiff University Reseach Ethics Committee) and the U.K. Animals (Scientific Procedure) Act, 1986 (Home Office Project License Number PPL 30/2413). All efforts were made to minimize the suffering and number of animals used in each experiment.

Breeding pairs of heterozygous SSADH deficient mice were obtained from Jackson Laboratories (Bar Harbor, ME, U.S.A). Offspring were genotyped as described previously [6], and experiments performed on postnatal day (P)23–31 SSADH^−/−^ and WT littermates. Despite initial reports of lethal status epilepticus occurring in SSADH^−/−^ mice from the end of the third postnatal week [6], [21], inbreeding of subsequent generations has ameliorated the severity of this phenotype so that mice are now capable of surviving into adulthood [23].

### Slice preparation and electrophysiology

Horizontal slices containing the VB thalamus and NRT, or coronal slices containing the somatosensory (barrel) cortex were prepared as described previously [26]. Briefly, male and female WT and SSADH^−/−^ mice were anaesthetised with isoflurane and decapitated. The brain was rapidly removed and slices cut in ice-cold, continuously oxygenated (95% O_2_: 5% CO_2_) artificial cerebrospinal fluid (aCSF) containing (in mM): NaCl 85, NaHCO_3_ 26, KCl 2.5, NaH_2_PO_4_ 1.25, MgCl_2_ 2, CaCl_2_ 2, glucose 10, sucrose 73.6, kynurenic acid 3, and indomethacin 0.045. Kynurenic acid and indomethacin were included in the cutting medium in order to improve slice viability [17]. Slices were incubated at room temperature in the above aCSF, but without kynurenic acid and indomethacin, for 15 mins before the sucrose-containing aCSF was gradually replaced over a period of 4–6 hrs with continuously oxygenated aCSF containing (in mM): NaCl 126, NaHCO_3_ 26, KCl 2.5, NaH_2_PO_4_ 1.25, MgCl_2_ 2, CaCl_2_ 2, and glucose 10. Slices were further incubated for at least 1 hr before being transferred to the recording chamber and perfused with warmed (33±1°C), continuously oxygenated aCSF containing (in mM): NaCl 126, NaHCO_3_ 26, KCl 2.5, NaH_2_PO4 1.25, MgCl_2_ 1, CaCl_2_ 2, glucose 10, and kynurenic acid 3.

Neurons were visualised using a Nikon microscope (Eclipse E600FN; Tokyo, Japan) equipped with a 40× immersion lens and a video camera (Hamamatsu, Hamamatsu City, Japan). The rodent VB thalamus and NRT contain a relatively uniform population of thalamocortical and GABAergic neurons, respectively [24], [25], whilst cortical layer 5/6 pyramidal neurons were identified by their characteristic shape and the presence of a large apical dendrite. Whole-cell patch clamp recordings were made using pipettes pulled from standard wall borosilicate glass (GC120F-10; Harvard Apparatus, Edenbridge, Kent, U.K.) attached to the headstage of a Multiclamp 700B amplifier controlled by Multiclamp Commander software (Molecular Devices, Sunnyvale, CA, U.S.A). Pipettes had a tip resistance of 2–4 MΩ when filled with solution containing (in mM): CsCl 130, MgCl_2_ 2, Mg-ATP 4, Na-GTP 0.3, HEPES 10, and EGTA 0.1; pH 7.25–7.30, ∼290 mOsm. Neurons were held at −70 mV, and since the reversal potential of Cl^−^ was ∼0 mV GABA_A_ currents appeared inward. Series resistance and whole-cell capacitance were determined in response to 5 mV voltage steps. Series resistance was compensated by ∼80%, and recordings discarded if it increased by >30%. Data were digitized at 20 kHz (Digidata 1322A, Molecular Devices), acquired using pClamp 9.0 software (Molecular Devices), and stored on a personal computer.

### Data analysis

Data were analysed as described previously [17], [26]. Briefly, data were filtered at 3 kHz and converted to an ASCII format for analysis using LabView based software (National Instruments, Austin, TX, U.S.A.). For analysis of spontaneous and miniature IPSCs, populations of individual IPSCs were averaged, and the peak amplitude, charge transfer (the integral of the average IPSC), weighted decay time constant (integral of the average IPSC divided by peak amplitude), frequency, and total current (charge transfer × frequency) measured. Tonic GABA_A_ currents were revealed as a shift in baseline current following the focal application of the GABA_A_ antagonist 6-imino-3-(4-methoxyphenyl)-1-(6*H*)-pyridazinebutanoic acid hydrobromide (gabazine). To measure tonic current amplitude, 5 ms epochs of baseline current were sampled every 100 ms, and those epochs that fell on IPSCs discarded. The average baseline current was then determined for two 5 s periods before gabazine application (i and ii) and one period after (iii). The background ‘drift’ of the baseline current was then calculated as the difference between the two pre-gabazine periods (i.e. ii-i), and the ‘shift’ in baseline current due to block of a tonic GABA_A_ current as the difference between the second pre-gabazine period and the post-gabazine period (i.e. iii-ii). A tonic current was presumed to be present for a given neuron if the post-gabazine shift was greater than twice the standard deviation of the pre-gabazine drift. The presence of tonic currents in a population of neurons (i.e. pre-gabazine drift *vs.* post-gabazine shift) was tested using Student's paired t-test, with significance set at P<0.05. Tonic current amplitude was also normalized to the whole-cell capacitance for each neuron.

To compare the post-natal age of WT and SSADH^−/−^ mice, absolute and normalized tonic current amplitudes, spontaneous and miniature IPSC properties between genotypes, and the effects of tetrodotoxin alone or together with (2*S*)-3-[[(1*S*)-1-(3,4-dichlorophenyl)ethyl]amino-2-hydroxypropyl](phenylmethyl)phosphonic acid (CGP55845) on tonic current amplitude and IPSC properties within genotypes, we used Student's unpaired t-test with significance set at P<0.05. Differences in the distribution of inter-IPSC intervals between genotypes were compared using the Kolmogorov-Smirnov test, with significance set at P<0.05. Data are presented as mean ± s.e.m.

Gabazine was focally applied to the slice using a pipette. Tetrodotoxin and CGP55845 were bath applied. CGP558945 was initially dissolved in DMSO before addition to the aCSF. Gabazine and tetrodotoxin were obtained from Ascent Scientific (Bristol, U.K.) and CGP55845 from Tocris (Bristol, U.K.).

## Results

Data were obtained from 19 WT and 14 SSADH^−/−^ mice. The mean age was not significantly different between the two genotypes (WT: 25.7±0.5 days, SSADH^−/−^: 25.0±0.5 days).

### Thalamocortical neurons

**Figure 1:**
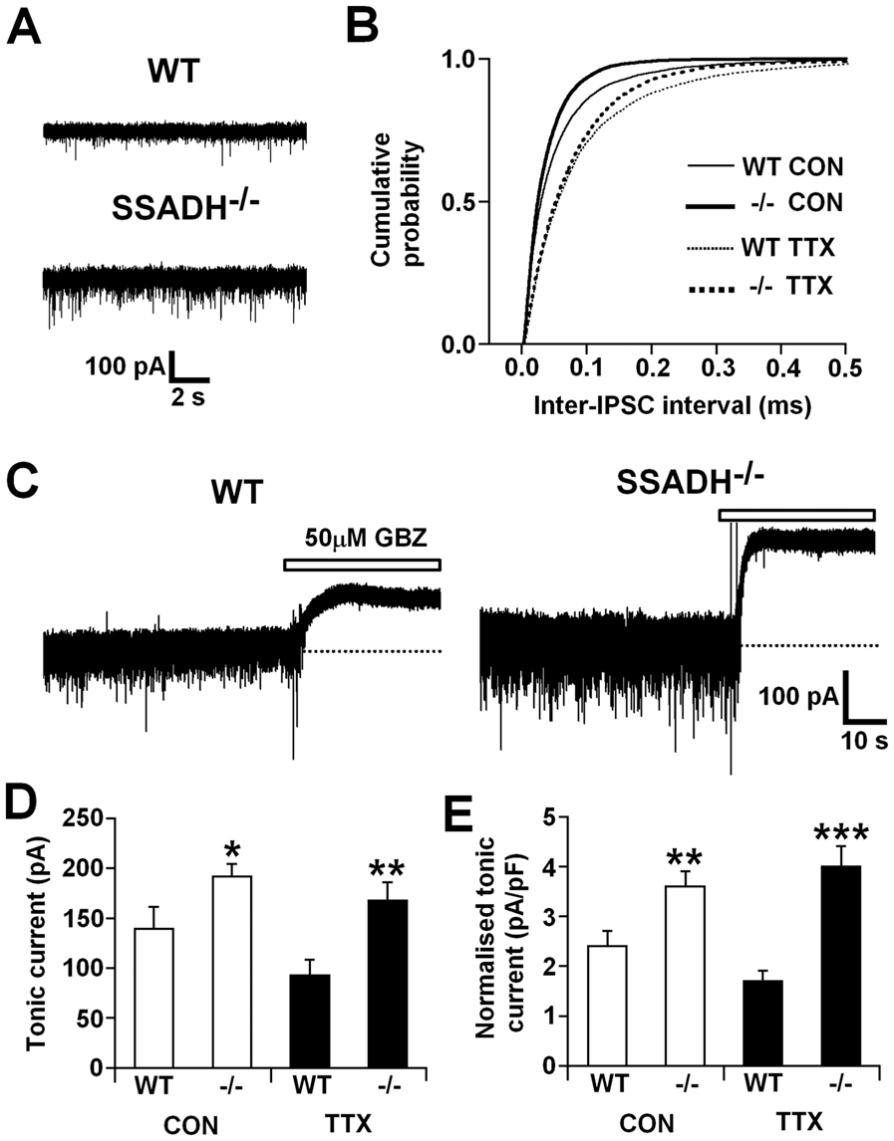
Increased tonic GABA currents and altered IPSC properties in TC neurons of SSADH^−/−^ mice. A, representative current traces from thalamocortical neurons of wild-type (WT, top) and SSADH^−/−^ (bottom) mice showing the differences in spontaneous IPSC frequency and amplitude between the two genotypes. B, cumulative probability plot showing the distribution of inter-IPSC intervals under control conditions (Con) (WT, thin line, 10678 inter-IPSC intervals; SSADH^−/−^, thick line, 11337 inter-IPSC intervals) and in the presence of 0.5 µM TTX (WT, thin dashed line, 5767 inter-IPSC intervals; SSADH^−/−^, thick dashed line, 7138 inter-IPSC intervals). C, representative current traces from thalamocortical neurons of WT (left) and SSADH^−/−^ (right) mice showing the difference in tonic current amplitude between the two genotypes. Dotted lines represent the initial baseline current prior to the focal application of 50 µM GBZ (white bar). D, graph comparing absolute tonic current amplitude in WT and SSADH^−/−^ mice under control conditions (Con, white columns), and in the presence of TTX (black columns). E, graph comparing normalized tonic current amplitude between WT and SSADH^−/−^ mice under control conditions (Con) and in the presence of TTX. D and E, * P<0.05, ** P<0.01 and *** P<0.001 WT *vs.* SSADH^−/−^, Student's unpaired t-test.

Under control conditions, i.e. in the presence of kynurenic acid (3 mM) to block ionotropic glutamate receptors and isolate GABA_A_ receptor mediated currents (Cope et al., 2009), sIPSCs were readily apparent in thalamocortical neurons of the VB thalamus from both WT (n = 11 cells) and SSADH^−/−^ (n = 9 cells) mice (Fig. 1A and Table 1), as described previously [17], [26], [27]. Comparison of sIPSC properties between WT and SSADH^−/−^ mice showed that peak amplitude, frequency, charge transfer and total current were significantly larger in SSADH^−/−^ mice (all P<0.05) (Table 1). Furthermore, there was a significant difference in the distribution of inter-IPSC intervals between WT and SSADH^−/−^ mice (P<0.001), so that shorter inter-IPSC intervals were more prevalant in SSADH^−/−^ mice (Fig. 1B). We also measured tonic GABA_A_ currents in the same thalamocortical neurons following the focal application of the GABA_A_ antagonist gabazine (GBZ, 50 µM) (Fig. 1C) [17], [26], [27]. Tonic currents were observed in every neuron recorded from both WT and SSADH^−/−^ mice, but were significantly larger in SSADH^−/−^ mice compared to WTs (WT: 139.2±21.6 pA, SSADH^−/−^: 191.5±12.1 pA; P<0.05) (Fig. 1C and D). Tonic current amplitude remained significantly larger in SSADH^−/−^ mice when normalized to whole-cell capacitance (WT: 2.4±0.3 pA/pF, SSADH^−/−^: 3.6±0.3 pA/pF; P<0.05) (Fig. 1E). Interestingly, in heterozygous animals (SSADH^+/−^) the tonic current in thalamocortical neurons was slightly, but not significantly (P>0.05), larger than WT animals but less than (P<0.05) that observed in SSADH^−/−^ (SSADH^+/−^: 147.1±19.8 pA; Normalised: 2.6±0.4 pA/pF). This finding is consistent with previously published data in dentate gyrus granule cells that also showed the magnitude of tonic GABAergic inhibition is dependent upon SSADH ‘gene dosage’ [28].

**Table 1:** Comparison of spontaneous and miniature IPSC properties, and the effects of CGP55845 on miniature IPSCs, in thalamocortical neurons of the ventrobasal thalamus from wildtype (WT) and SSADH^−/−^ mice.

IPSC parameter						
	*n*	Peak amplitude(pA)	Weighted decay(ms)	Frequency(Hz)	Charge transfer(fC)	Total current(pA)
**sIPSCs**						
**WT**	(11)	−52.5±3.2	1.8±0.1	18.5±3.6	−106.9±7.7	−1.9±0.4
**SSADH^−/−^**	(9)	−83.9±4.7***	1.6±0.1	33.6±6.6*	−162.3±15.7**	−5.4±1.1**
**mIPSCs**						
**WT**	(10)	−61.5±4.1	1.4±0.1†††	10.5±2.2	−99.5±8.3	−1.1±0.3
**SSADH^−/−^**	(10)	−73.5±7.6	1.4±0.1	13.0±1.3††	−120.2±13.7†	−1.6±0.2††
**mIPSCs + CGP55845**						
**WT**	(11)	−57.7±3.8	1.6±0.1‡	10.4±1.4	−104.2±7.9	−1.2±0.2
**SSADH^−/−^**	(7)	−77.6±13.5	1.7±0.2	8.5±1.8‡	−167.5±49.0	−1.3±0.3

Data are presented as mean ± s.e.m.

*P<0.05,

**P<0.01 and

***P<0.001, WT *vs.* SSADH^−/−^.

† P<0.05,

†† P<0.01 and

††† P<0.001, sIPSCs *vs.* mIPSCs within genotypes.

‡ P<0.05, mIPSCs *vs.* mIPSCs+CGP55845 within genotypes. Number of recorded neurons (***n***) is as indicated.

We also measured mIPSC properties in thalamocortical neurons following bath application of tetrodotoxin (TTX, 0.5 µM). Comparison of mIPSC properties between WT (n = 10 cells) and SSADH^−/−^ (n = 10 cells) mice revealed no significant differences (Table 1), although the distribution of inter-IPSC intervals was significantly different between the two genotypes (P<0.001), indicating a trend for a higher mIPSC frequency in SSADH^−/−^ animals (Fig. 1B). In both WT and SSADH^−/−^ mice there was a general trend for the frequency of mIPSCs to be reduced compared to sIPSCs, but this was only significant for SSADH^−/−^ mice (SSADH^−/−^: P<0.01; WT: P = 0.07), and for the weighted decay time constant to be faster, but this was only significant for WT mice (Table 1). In addition the charge transfer and total current of mIPSCs was significantly smaller in SSADH^−/−^ mice compared to sIPSCs (P<0.05 and P<0.01, respectively) (Table 1). Tonic current amplitude in the same thalamocortical neurons was also reduced in both strains compared to control conditions (WT: 92.8±15.1 pA, SSADH^−/−^: 167.5±17.9 pA), but not significantly (Fig. 1D). Similarly, the normalized tonic current amplitude in both genotypes was also not significantly different compared to control conditions (WT: 1.7±1.0 pA/pF, SSADH^−/−^: 4.0±0.4 pA/pF) (Fig. 1E). However, both absolute and normalized tonic current amplitude were still significantly larger in SSADH^−/−^ compared to WT mice in the presence of TTX (absolute P<0.01, normalized P<0.001) (Fig. 1D and E). Thus, increased phasic and tonic GABA_A_ receptor-mediated inhibition occurs in thalamocortical neurons of the somatosensory thalamus from SSADH^−/−^ mice.

### NRT neurons

**Figure 2:**
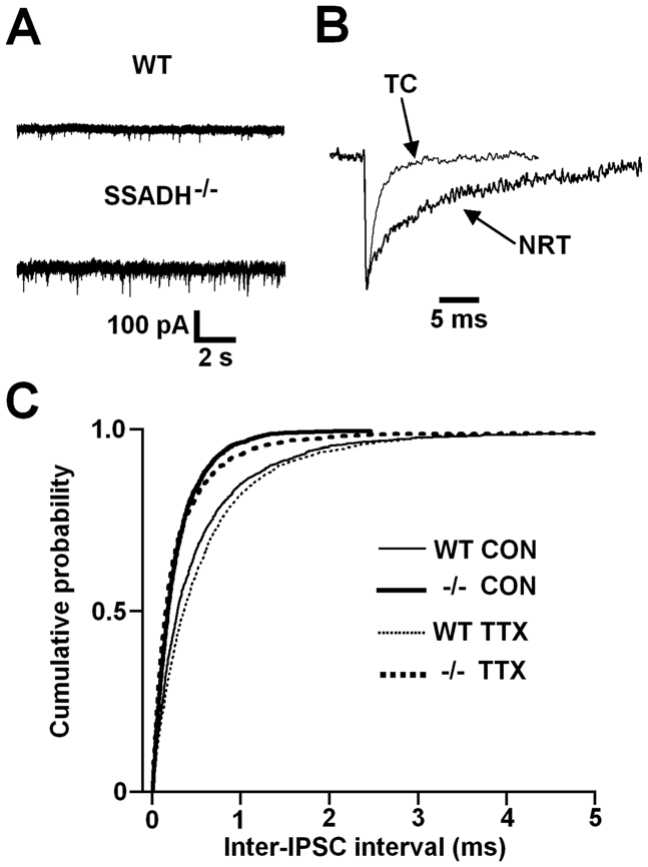
Increased IPSC frequency in NRT neurons of SSADH^−/−^ mice. A, representative current traces from NRT neurons of wild-type (WT, top) and SSADH^−/−^ (bottom) mice showing the difference in spontaneous IPSC frequency between the two genotypes. B, the waveforms of average spontaneous IPSCs from a thalamocortical (TC) neuron of the VB thalamus and an NRT neuron. Note the characteristically slower decay of the IPSC from the NRT neuron compared to the thalamocortical neuron. IPSCs have been normalized to the same peak amplitude. C, cumulative probability plot showing the distribution of inter-IPSC intervals under control conditions (Con) (WT, thin line, 1936 inter-IPSC intervals; SSADH^−/−^, thick line, 1710 inter-IPSC intervals) and in the presence of 0.5 µM TTX (WT, thin dashed line, 1759 inter-IPSC intervals; SSADH^−/−^, thick dashed line, 2143 inter-IPSC intervals).

The frequency of sIPSCs in NRT neurons from both WT and SSADH^−/−^ mice was lower compared to thalamocortical neurons (compare Figs. 1A and 2A, and Tables 1 and 2), in agreement with previous studies in rodents [26], [27], [29], [30]. Furthermore, sIPSCs in NRT neurons exhibited a characteristically slower decay compared to those in thalamocortical neurons (Fig. 2B). Comparison of NRT neuron sIPSC properties between WT and SSADH^−/−^ mice (n = 9 and 6 cells, respectively) showed that sIPSC frequency and total current were significantly larger in SSADH^−/−^ mice (both P<0.05) (Fig. 2A and Table 2). In agreement with this, the distribution of inter-IPSC intervals between WT and SSADH^−/−^ mice was significantly different (P<0.001) so that shorter inter-IPSC intervals were more apparent in SSADH^−/−^ mice (Fig. 2C). Similar to thalamocortical neurons, we focally applied GBZ to NRT neurons of both genotypes (n = 3 cells each), to test for the presence of tonic currents. However, tonic currents were never observed (data not shown), in agreement with previous studies in NRT neurons [26], [27].

**Table 2:** Comparison of spontaneous and miniature IPSC properties in NRT neurons of wildtype (WT) and SSADH^−/−^ mice.

IPSC parameter						
	*n*	Peak amplitude(pA)	Weighted decay(ms)	Frequency(Hz)	Charge transfer(fC)	Total current(pA)
**sIPSCs**						
**WT**	(9)	−36.4±3.2	12.3±0.4	1.8±0.4	−456.8±44.5	−0.9±0.3
**SSADH^−/−^**	(6)	−43.2±3.1	12.7±0.4	3.5±0.6*	−559.3±34.9	−2.0±0.5*
**mIPSCs**						
**WT**	(9)	−40.7±2.9	12.8±0.7	1.6±0.2	−525.8±39.1	−0.9±0.1
**SSADH^−/−^**	(6)	−35.5±3.2	12.5±0.8	3.9±1.5	−443.8±23.8†	−1.8±0.7

Data are presented as mean ± s.e.m.

*P<0.05, WT *vs.* SSADH^−/−^.

† P<0.05, sIPSCs *vs.* mIPSCs within genotypes. Number of recorded neurons (***n***) is as indicated.

In the presence of 0.5 µM TTX, the parameters of mIPSCs recorded from NRT neurons were not significantly different between WT and SSADH^−/−^ mice (n = 9 and 6 cells, respectively). However, there was a clear trend for mIPSC frequency to be greater in SSADH^−/−^ mice compared to WTs (P = 0.07), that was reflected in the significantly different distribution of inter-IPSC intervals between genotypes (P<0.001) (Fig. 2C). There were no differences in the properties of sIPSCs and mIPSCs in WT mice, but mIPSCs in SSADH^−/−^ mice had a significantly smaller charge transfer compared to sIPSCs (P<0.05) (Table 2). Since we did not observe tonic currents in NRT neurons under control conditions, we did not test for their presence following the application of TTX. These results demonstrate increased phasic GABA_A_ inhibition in NRT neurons of SSADH^−/−^ mice compared to WT littermates.

### Layer 5/6 pyramidal neurons

**Figure 3:**
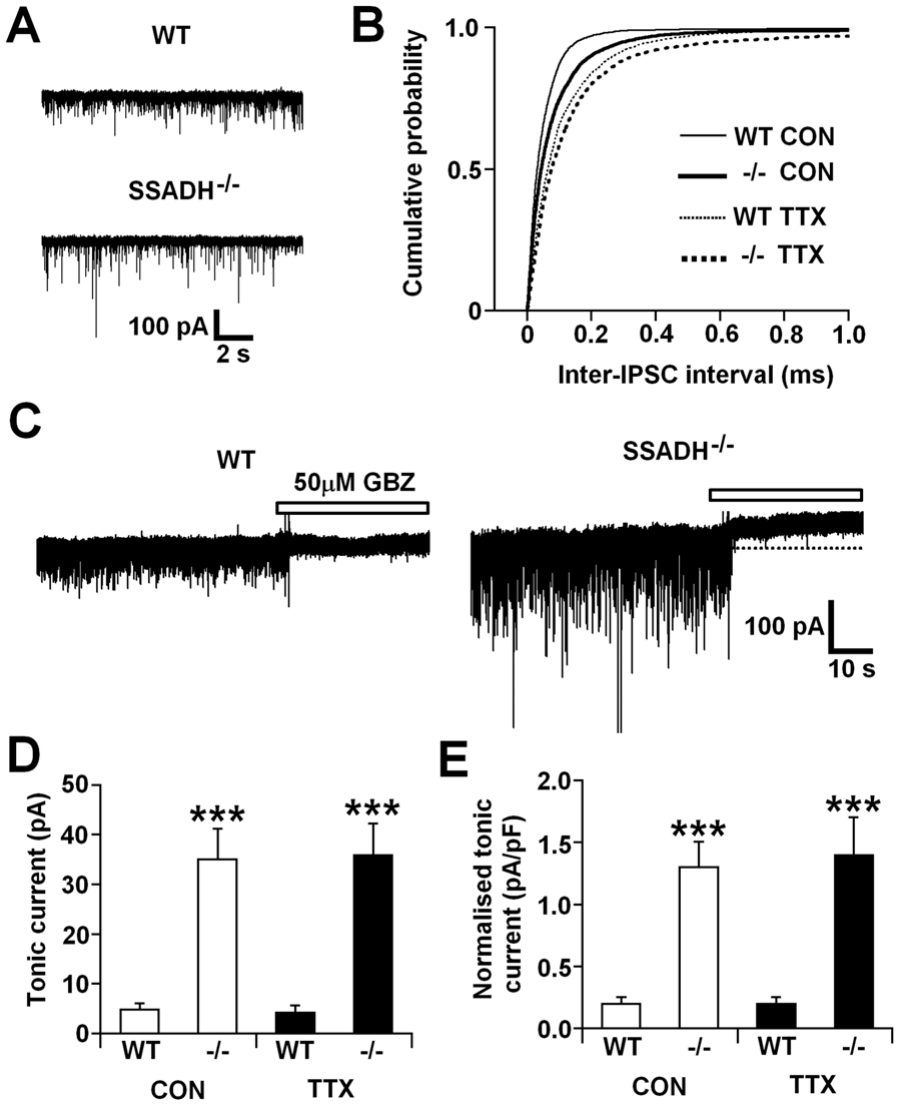
Increased tonic GABA current and IPSC frequency in layer V cortical neurons in SSADH^−/−^ mice. A, representative current traces from layer 5/6 pyramidal neurons of wild-type (WT, top) and SSADH^−/−^ (bottom) mice showing the difference in spontaneous IPSC amplitude between the two genotypes. B, cumulative probability plot showing the distribution of inter-IPSC intervals under control conditions (Con) (WT, thin line, 9356 inter-IPSC intervals; SSADH^−/−^, thick line, 6245 inter-IPSC intervals) and in the presence of 0.5 µM TTX (WT, thin dashed line, 3430 inter-IPSC intervals; SSADH^−/−^, thick dashed line, 2977 inter-IPSC intervals). C, representative current traces from layer 5/6 pyramidal neurons of WT (left) and SSADH^−/−^ (right) mice showing the difference in tonic current amplitude between the two genotypes. Note that a tonic current is not apparent in the neuron from the WT animal. Dotted lines represent the initial baseline current prior to the focal application of 50 µM GBZ (white bar). D, graph comparing absolute tonic current amplitude in WT and SSADH^−/−^ mice under control conditions (Con, white columns), and in the presence of TTX (black columns). E, graph comparing normalized tonic current amplitude between WT and SSADH^−/−^ mice under control conditions (Con) and in the presence of TTX. D and E, *** P<0.001 WT *vs.* SSADH^−/−^, Student's unpaired t-test.

Comparison of sIPSCs in layer 5/6 pyramidal neurons of the somatosensory cortex from WT (n = 9 cells) and SSADH^−/−^ (n = 9 cells) mice revealed a significantly larger peak amplitude and charge transfer in SSADH^−/−^ mice (Fig. 3A and Table 3) (P<0.001 and P<0.05, respectively), and although there was no difference in sIPSC frequency between genotypes, the distribution of inter-IPSC intervals was significantly different (P<0.001) in favour of shorter inter-IPSC intervals in WT mice (Fig. 3B). In the same neurons from WT mice, focal application of GBZ failed to reveal a tonic current (absolute 4.8±1.2 pA, normalized 0.2±0.1 pA/pF) (Fig. 3C–E), as described previously for layer 2/3 pyramidal neurons in these WT mice [21]. In contrast, focal application of GBZ in SSADH^−/−^ mice revealed robust tonic currents (absolute 35.0±6.1 pA, normalized 1.3±0.2 pA/pF) (Fig. 3C) that were significantly larger compared to WT mice (absolute and normalized, both P<0.001) (Fig. 3D and E).

**Table 3:** Comparison of spontaneous and miniature IPSC properties in somatosensory cortical layer 5/6 pyramidal neurons from wildtype (WT) and SSADH^−/−^ mice.

IPSC parameter	*n*	Peak amplitude(pA)	Weighted decay(ms)	Frequency(Hz)	Charge transfer(fC)	Total current(pA)
**sIPSCs**						
**WT**	(9)	−62.4±5.3	2.4±0.2	18.9±3.2	−166.4±23.8	−3.2±0.7
**SSADH^−/−^**	(9)	−96.9±6.4***	2.6±0.4	12.6±3.0	−261.9±28.3*	−2.8±0.4
**mIPSCs**						
**WT**	(7)	−70.4±5.5	2.1±0.3	8.9±2.2†	−164.6±28.2	−1.4±0.3†
**SSADH^−/−^**	(7)	−89.4±8.0*	3.0±0.5	7.8±1.9	−286.9±42.4*	−2.0±0.5

Data are presented as mean ± s.e.m.

*P<0.05 and

***P<0.001, WT *vs.* SSADH^−/−^.

† P<0.05, sIPSCs *vs.* mIPSCs within genotypes. Number of recorded neurons (*n*) is as indicated.

In the presence of TTX, the peak amplitude and charge transfer of mIPSCs recorded from layer 5/6 pyramidal neurons were larger in SSADH^−/−^ mice (n = 7 cells) compared to WT mice (n = 7 cells) (both P<0.05) (Table 3), and the distribution of inter-IPSC intervals was also significantly different (P<0.001), indicating shorter inter-IPSC intervals in WT mice (Fig. 3B). In WT mice, the frequency and total current of mIPSCs were significantly smaller compared to sIPSCs (both P<0.05), but there was no difference in the properties of mIPSCs and sIPSCs in SSADH^−/−^ mice (Table 3). Tonic currents in WT mice were still not apparent in the presence of TTX (absolute 4.3±1.3 pA, normalized 0.2±0.1 pA/pF) (Fig. 3D and E), and TTX had no effect on absolute and normalised tonic current amplitude in SSADH^−/−^ mice compared to control conditions (absolute 35.8±6.3 pA, normalized 1.4±0.3 pA/pF) (Fig. 3D and E). However, absolute and normalized tonic current amplitudes in the presence of TTX remained significantly larger in SSADH^−/−^ mice compared to WTs (both P<0.001). Thus, both spontaneous and miniature IPSCs are larger, but fewer, in layer 5/6 pyramidal neurons of SSADH^−/−^ mice compared to WTs, and SSADH^−/−^ mice also exhibit larger tonic currents.

### Block of GABA_B_ receptors reduces tonic current amplitude in TC neurons of SSADH^−/−^ but not WT mice

**Figure 4:**
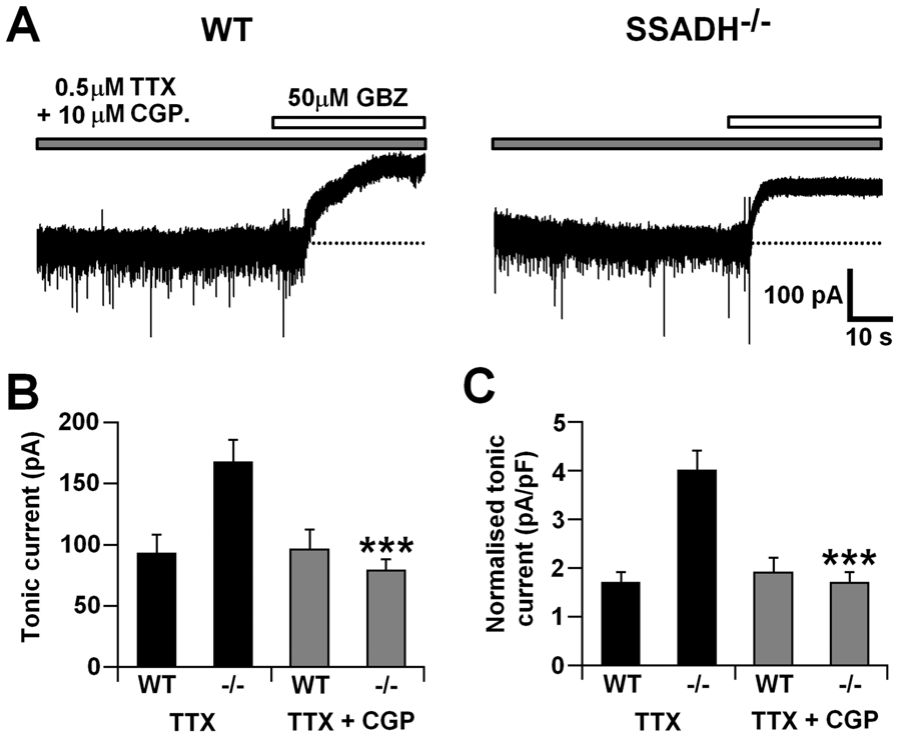
Contribution of GABA_B_ receptors to enhanced tonic inhibition in TC neurons of SSADH^−/−^ mice. A, representative current traces from TC neurons of wild-type (WT, left) and SSADH^−/−^ (right) mice in the continuing presence of 0.5 µM TTX+10 µM CGP55845 (grey bar). Note the tonic current is smaller in the neuron from the SSADH^−/−^ mouse compared to the WT mouse. Dotted lines represent the initial baseline current prior to the focal application of 50 µM GBZ (white bar). B, graph comparing absolute tonic current amplitude in WT and SSADH^−/−^ mice in the presence of TTX alone (TTX, black columns), and in the presence of TTX+CGP55845 (TTX+CGP., grey columns). C, graph comparing normalized tonic current amplitude in WT and SSADH^−/−^ mice in the presence of TTX alone (TTX) and in the presence of TTX+CGP55845 (TTX+CGP.). B and C, *** P<0.001 TTX *vs.* TTX+CGP. within genotypes, Student's unpaired t-test.

Previously, we described a role for GABA_B_Rs in facilitation of tonic eGABA_A_R currents in TC neurons of several animal models of typical absence seizures including the Genetic Absence Epilsepy Rat from Strasbourg (GAERS) and stargazer and lethargic mice [17]. As SSADH deficiency leads not only to increased GABA concentration in the CSF but also increased GHB (a GABA_B_R agonist) levels we tested the contribution of GABA_B_Rs to the enhancement of tonic GABA_A_ current observed in TC neurons of SSADH^−/−^ mice. In the presence of TTX the GABA_B_R antagonist CGP55845 (10 µM) produced a significant reduction in mIPSC frequency in VB TC neurons from SSADH^−/−^ mice but not in those from WT mice (P<0.01) (Table 1) without significant (P>0.05) differences in amplitudes of synaptic currents in either mouse. In WT mice CGP55845 also resulted in a significant increase (P<0.01) in the weighted decay time of mIPSCs, a trend which was also observed in SSADH^−/−^ mice (although differences were not significant, P>0.05). Importantly, in the same neurons CGP55845 caused a significant reduction in the enhanced tonic current observed in SSADH^−/−^ mice (TTX: 167.5±17.9 pA, n = 10; TTX+CGP: 74.3±8.5 pA, n = 7; P<0.001) but not in WT littermates (TTX: 92.9±15.1 pA, n = 10; TTX+CGP: 96.5±15.7 pA, n = 11; P>0.05) (Fig. 4). These findings indicated that in SSADH^−/−^ mice GABA_B_R-dependent modulation of tonic GABA_A_ currents in VB TC neurons may contribute to the observed absence epilepsy phenotype as has been demonstrated in other models of this pathological state.

## Discussion

Typical absence seizures are a clinical phenotype of many idiopathic generalized epilepsies, and although several GABA_A_ receptor subunit mutations in cohorts of patients with absence have been identified [31]–[34], and a causative role of compromised GABA_A_ receptor-mediated inhibition in seizure genesis has been suggested [15], [16], it is apparent that GABA_A_ receptor gain-of-function in cortico-thalamic networks may be an underlying mechanism for these seizures [17], [35]–[37]. The data presented here support these findings by identifying increased phasic and tonic GABA_A_ inhibition in the principal cell types involved in the generation of typical absence seizures from SSADH^−/−^ mice, a rodent model of SSADH deficiency. In thalamocortical neurons of SSADH^−/−^ mice, sIPSC frequency and peak amplitude were greater, and in NRT neurons sIPSC frequency was also greater, leading to increased total phasic current in both cell types. Block of action potential-dependent GABA release by TTX effectively normalised IPSC properties in both thalamocortical and NRT neurons so that mIPSCs in SSADH^−/−^ mice were no different to those in WTs. Therefore increased phasic inhibition in thalamic neurons of SSADH^−/−^ mice appears to be largely driven by enhanced activity-dependent GABA release. However, in both thalamocortical and NRT neurons, mIPSC frequency, as determined by the distribution of inter-IPSC intervals, was indeed greater in SSADH^−/−^ mice, perhaps also indicating an activity-independent increase in vesicular GABA release. This is of particular interest since NRT neurons are the primary source of GABAergic input to the rodent VB thalamus [24], [38] and also innervate one another [39] but whereas GABAergic synapses in the VB thalamus are axo-dendritic, GABAergic synapses in the NRT are dendro-dendritic [39]. Therefore, despite this fundamental difference in synapse physiology, both types of synapse appear to share GABA release mechanisms that are altered in SSADH^−/−^ mice, perhaps preferentially at dendro-dendritic synapses. In layer 5/6 pyramidal neurons, there was a clear trend for spontaneous and miniature IPSC frequencies to be lower in SSADH^−/−^ mice compared to WTs, as measured by the distribution of inter-IPSC intervals, but the peak amplitude of both sIPSCs and mIPSCs was significantly larger in the mutant mice, effectively negating the change in frequency so that total phasic current was no different between genotypes. The increased peak amplitude in the presence of TTX therefore indicates that GABA_A_ receptor number is increased in layer 5/6 pyramidal neurons, perhaps to counteract a reduction in activity-dependent and -independent vesicular GABA release. As well as aberrant phasic inhibition, we also observed a clear increase in tonic currents in thalamocortical and layer 5/6 pyramidal neurons of SSADH^−/−^ mice compared to WTs. This is not surprising since GABA levels are approximately 3 fold higher in the cerebrospinal fluid of SSADH^−/−^ mice [6]. Given our recent findings in normal Wistar rats [17], we also suggest that elevated GHB levels, approximately 40 fold in SSADH^−/−^ mice, may contribute to increased tonic inhibition.

Previous studies in SSADH^−/−^ mice identified no changes in either spontaneous or evoked IPSCs in layer 2/3 pyramidal neurons, but did demonstrate a larger tonic current in these neurons [21], whilst mIPSC frequency and GABA_A_ IPSPs were reduced in CA1 pyramidal neurons [20], [22]. The loss of GABA_A_ inhibition in CA1 pyramidal neurons is in good agreement with reduced [^35^S]tert-butylbicyclophosphorothionate ([^35^S]TBPS) binding in the hippocampus of SSADH^−/−^ mice and a reduction of β2 subunit, but not α1, β3 or γ2 subunits, expression [20], [22]. However, reduced [^35^S]TBPS was also observed in both the cortex and thalamus of SSADH^−/−^ mice, but our data and those of Drasbek et al. (2008) indicate that GABA_A_ receptor function is either unaltered or increased in these areas. One possible explanation is that the loss of GABA_A_ receptors in the cortex and thalamus is an attempt to homeostatically compensate for increased levels of GABA and GHB, which is ultimately unsuccessful. However, it must also be born in mind that GABA_A_ receptor expression as measured by [^3^H]muscimol or [^3^H]flunitrazepam binding was no different in SSADH^−/−^ and WT mice [20]. In any case, taken together, these studies highlight how changes in GABA_A_ inhibition in the same model of epilepsy may be cell-type specific, as evidenced by enhanced tonic currents in thalamocortical neurons, but reduced tonic currents in dentate gyrus granule cells, of stargazer mice [17], [40].

We previously demonstrated enhanced tonic inhibition in thalamocortical neurons from diverse genetic and pharmacological models of absence seizures [17], and these findings are extended by our present observations in thalamocortical neurons of SSADH deficient mice. Extrasynaptic GABA_A_ receptors in thalamocortical neurons are critical for seizure genesis in two of the best established models of absence, the genetic absence epilepsy rats from Strasbourg (GAERS) and GHB models, and their selective activation can induce seizures in normal animals [17]. It therefore seems likely that increased tonic GABA_A_ inhibition in thalamocortical neurons is also important for the appearance of seizures in SSADH deficient mice. However, the pathological consequences of enhanced phasic inhibition in thalamic neurons remains to be determined, although increased frequency and peak amplitude of mIPSCs and loss of IPSC paired-pulse depression has been documented in NRT neurons of GAERS [41], that may lead to increased excitability and hypersynchrony [42]. This does not explain, though, why the mode of action of clonazepam, a frontline treatment for absence epilepsy, is to selectively increase phasic inhibition in NRT neurons [43]. Furthermore, the pro-epileptic role of aberrant phasic and tonic inhibition, if any, in layer 5/6 cortical neurons of SSADH^−/−^ mice, or indeed other models of typical absence seizures, also remains to be elucidated.

In summary, SSADH deficient mice exhibit synaptic and extrasynaptic GABA_A_ receptor gain-of-function in cortico-thalamic networks that may underlie the appearance of typical absence seizures. Such gain-of-function appears to be a defining feature of typical absence seizures, and may have pathological consequences in patients deficient for SSADH.
